# Invasive plants may promote predator-mediated feedback that inhibits further invasion

**DOI:** 10.1002/ece3.1525

**Published:** 2015-05-25

**Authors:** Lauren M Smith, Oswald J Schmitz

**Affiliations:** 1 Yale University School of Forestry and Environmental Studies370 Prospect Street, New Haven, Connecticut, 06510; 2 Yale University School of Forestry and Environmental Studies195 Prospect Street, New Haven, Connecticut, 06510

**Keywords:** *Alliaria petiolata*, biotic resistance, enemy escape, food web, garlic mustard, model, negative feedback, plant invasion, predator-mediation, spiders, Theridiidae, top-down control

## Abstract

Understanding the impacts of invasive species requires placing invasion within a full community context. Plant invaders are often considered in the context of herbivores that may drive invasion by avoiding invaders while consuming natives (enemy escape), or inhibit invasion by consuming invaders (biotic resistance). However, predators that attack those herbivores are rarely considered as major players in invasion. Invasive plants often promote predators, generally by providing improved habitat. Here, we show that predator-promoting invaders may initiate a negative feedback loop that inhibits invasion. By enabling top-down control of herbivores, predator-promoting invaders lose any advantage gained through enemy escape, indirectly favoring natives. In cases where palatable invaders encounter biotic resistance, predator promotion may allow an invader to persist, but not dominate. Overall, results indicate that placing invaders in a full community context may reveal reduced impacts of invaders compared to expectations based on simple plant–plant or plant–herbivore subsystems.

## Introduction

Placing invasive species within the context of ecological communities is essential to understand invasion mechanisms and predict invader impacts (Shea and Chesson 2002; White et al. 2006). To this end, past efforts have focused on direct plant–plant (Hierro and Callaway 2003; Levine et al. 2003), plant–soil (Inderjit and van der Putten 2010), and plant–herbivore (Keane and Crawley 2002) interactions. However, invasive plants may also interact with native predators, often elevating local predator densities (Pearson 2009, 2010; DeVore and Maerz 2014; Loomis et al. 2014) by enhancing predator habitat (Pearson 2009, 2010; Loomis et al. 2014) or improving conditions for predator survival (DeVore and Maerz 2014). This may precipitate indirect, cascading effects throughout the entire community whenever predators directly control the natural enemies of plants – herbivores – and thereby indirectly alleviate plant damage (White et al. 2006). The cascading effect in turn may lead to complex feedbacks that change the dynamics and relative success of the invasive plant itself. Yet, this potential, and its relative importance in invasion dynamics, remains altogether underexplored (White et al. 2006). Here, we advance understanding of such feedbacks through theoretical analysis exploring how promotion of predators by invasive plants has community-level effects that alter invasion dynamics.

The idea that invasive plants might alter community dynamics through feedbacks involving predators and the invading plant's natural enemies may have implications for one of the most commonly invoked invasion mechanisms: the “enemy escape” hypothesis. Invasive plants are routinely thought to escape herbivory by being unpalatable to herbivores relative to native plant species (Keane and Crawley 2002). This would put native plants at a competitive disadvantage because they experience greater losses to generalist herbivores than invaders do and must cope with damage from herbivory as well as competition in order to thrive. Evidence from the literature suggests that invaders tend to support lower abundance and diversity of generalists insects (Burghardt and Tallamy 2013), although experiments directly testing the role of this phenomenon in driving invasion have yielded mixed results (Keane and Crawley 2002). Empirical synthesis indicates that support for the enemy escape hypothesis comes mostly from observational studies rather than from direct experimental manipulation of natural enemies native to the invaded range. For example, observations of successful biocontrol efforts are often used as evidence of enemy escape, even though showing that introducing a specialist can reduce invader density is not the same as showing that release from that specialist caused the invasion. Furthermore, experimental evidence suggests that enemy escape is not universal, as some invaders receive equal or greater herbivore damage than co-occurring natives (Keane and Crawley 2002). This results in a need to explain why this mechanism sometimes fails to explain invasions. Considering enemy escape in a broader community context that includes predators may help to explain why the mechanism alone might not generally explain invader success. In that broader context, predator promotion by the invasive may lead to top-down control of herbivores that may attenuate damage to native plants. This would reduce the invader's advantage, potentially causing a negative feedback that inhibits invasion success.

Predator promotion may also have important implications for a hypothesis explaining how native communities resist invasion: the “biotic resistance” hypothesis. Under biotic resistance, native herbivores control highly palatable invaders (Maron and Vila 2001; Levine et al. 2004). In the face of strong biotic resistance, predator promotion may benefit an invader. A predator-promoting invasive plant might overcome biotic resistance by initiating predator control of native herbivores, resulting in “predator-mediated enemy escape.”

Invasive plants that promote predators, particularly spiders, do so by increasing the structural complexity of a habitat, thereby providing substrates for web-building spiders that are normally limited by availability of sites for building webs (Rypstra 1983). Invasive knapweed (*Centaurea stoebe*) (Pearson 2009), bush honeysuckle (*Lonicera maackii*) (Loomis et al. 2014), and garlic mustard (*Alliaria petiolata*) (Appendix A1) all support elevated densities of web-building spiders this way. Knapweed can cause a striking 38-fold increase in web-spider densities relative to native vegetation, and its physical structure facilitates web-building spiders to build larger webs and capture double the number of prey than captured when webs are erected on native vegetation (Pearson 2009). Web-building spiders were 2× as abundant in areas dominated by bush honeysuckle (Loomis et al. 2014) and 5× as abundant in areas invaded by garlic mustard (Appendix A1) compared to native vegetation. Invaders may improve habitat conditions for active-hunting spiders as well. The invasive grass *Microstegium vimineum* and the vine *Vinca minor* promote elevated densities of Lycosid (wolf) spiders by reducing cannibalism and predation among closely related spider species (Bultman and DeWitt 2008; DeVore and Maerz 2014). Predator promotion can lead to myriad direct effects on herbivorous insect communities (Pearson 2009) and co-occurring predator populations (DeVore and Maerz 2014). There are hints that the effects may even extend indirectly to affect the fecundity of neighboring plants (Pearson 2010). The broader implications for community dynamics remain unexplored.

**Figure 1:**
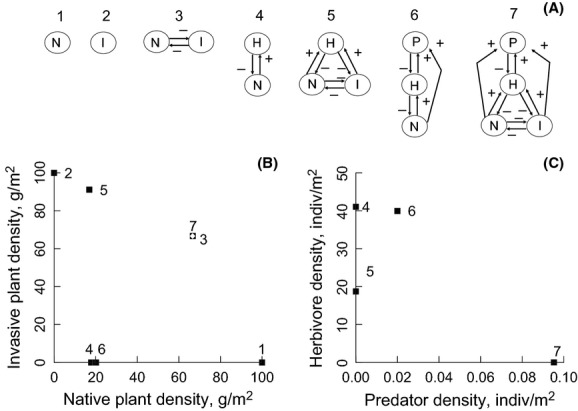
(A) Feasible subsets (1–6) and the complete model (7) were analyzed and compared to understand the role of predator promotion in a system with an invasive plant (I), a native plant (N), an herbivore (H), and a predator (P). Plots show equilibrium densities of (B) the native and invasive plant, and (C) the predator and herbivore for all species subsets. Point 3 (the two plants in competition) and point 7 (the full four-species system with invader, native, herbivore, and predator) overlap significantly in (B), so 7 is indicated by a white diamond. The predator and invader interact to promote elevated density of the native plant (7) compared to subsystems where the invader (4) or predator (6) is absent. While the herbivore appears to be extinct in point 7, it is actually at a very low equilibrium density of 0.014 indiv/m^2^, allowing it to support a high predator density (C).

Here, we report on an analysis exploring the potential for predators to mediate invasion dynamics using a food web model. We used the model to quantify the net effects of direct and indirect interactions and feedbacks of predator-promoting invaders on a community comprised of an invasive and native plant and herbivores. Our goal was to resolve whether the net effect of predator promotion by invaders is sufficiently large to reverse invasion dynamics, as verbally hypothesized. Predator mediation is not guaranteed. Theoretically, there is potential for the top-down effect to attenuate given food web complexity arising from the direct and indirect linkages among the community members (Fig.1A).

We consider two mechanisms that are broadly applicable to invasion ecology: (1) Unpalatable, predator-promoting invasive plants may reverse enemy escape through “predator-mediated negative feedback.” This arises because invaders that promote predators may inhibit their own success by reducing herbivory on native competitors; and (2) palatable invaders that are preferred by herbivores may overcome biotic resistance through “predator-mediated enemy escape.” This arises because invaders that promote predators reduce their own susceptibility to herbivory. Our modeling reveals the conditions under which these two mechanisms may become prevalent.

Our analysis, while general, is inspired by the case of the invading herb garlic mustard (*Alliaria petiolata*), a member of the Brassicaceae that was introduced from Eurasia to eastern North American deciduous forests. Like many successful invaders, it experiences reduced herbivory compared to natives due to its arsenal of chemical defenses (Rodgers et al. 2008). Garlic mustard plants develop dry dehiscent fruit structures that serve as ideal web substrates, resulting in elevated cobweb-spider densities where garlic mustard invades (Appendix A1). Because garlic mustard is an ideal case study to motivate our analysis, it shares characteristics of enemy escape and predator promotion with many other invaders.

We consider the dynamics of populations of the invasive plant, native plant, herbivores, and predators configured into an interdependent food web that combines important elements such as direct plant–plant competitive and herbivore–plant consumptive interactions that were previously examined independently (Keane and Crawley 2002; Hierro and Callaway 2003; Levine et al. 2003). Our food web model strikes a balance between having a sufficient level of complexity to examine community-level feedbacks, yet being simple enough to allow tractable analysis of how different mechanisms precipitate the feedbacks. We systematically examined how greater complexity led to feedbacks by comparing different species subsets. The analysis reveals that community-level feedback caused by predator promotion by invasive plants reduces impacts of invaders compared to expectations based on simple plant–plant or plant–herbivore systems. These results may explain why enemy escape often fails as an invasion mechanism (Keane and Crawley 2002), or why initially successful invaders may tend to decline over time, a phenomenon often attributed to enemy accumulation (Dostal et al. 2013; Flory and Clay 2013), habitat alteration (Tang et al. 2012), or losses of natural enemies of native species to disturbance (Roy et al. 2014) (Table1).

**Table 1:** State variables and parameters used in the food web model. Default values were estimated from the cited sources or from preliminary data. Explanations of estimates and data are available in Appendix A1

State Variable	Definition	Units
*V*_*N*_	Native vegetation	g/m^2^
*V*_*I*_	Invasive vegetation	g/m^2^
*H*	Herbivore	indiv/m^2^
*P*	Predator	indiv/m^2^
*t*	Time	days

Parameter	Definition	Units	Default value	Source
*r*_*N*_	Intrinsic growth rate, native plant	/day	0.1	Smith and Reynolds (2014)
*c*_*N*_	Strength of density dependence, native plant	/(g/m^2^)/day	0.001	Smith and Reynolds (2014)
*f*_*NH*_	Attack rate of herbivore on native plant	/indiv/day	0.002	Llewellyn (1970)
*r*_*I*_	Intrinsic growth rate, garlic mustard	/day	0.1	Smith and Reynolds (2014)
*c*_*I*_	Strength of density dependence, garlic mustard	/(g/m^2^)/day	0.001	Smith and Reynolds (2014)
*f*_*IH*_	Attack rate of herbivore on garlic mustard	/indiv/day	0.00002	Appendix A1
*a*	Ratio inter- to intraspecific competition (effect of I on N)	–	0.5	Appendix A1
*b*	Ratio inter- to intraspecific competition (effect of N on I)	–	0.5	Appendix A1
*g*_*H*_	Conversion efficiency of herbivore	indiv/g	1.4	Llewellyn (1972, 1975)
*m*_*H*_	Background mortality of herbivore	/day	0.05	Banks et al. (2008)
*f*_*P*_	Attack rate of predator on herbivore	/indiv/indiv/day	16	Sigsgaard et al. (2001)
*h*_*P*_	Half-saturation constant for predator	indiv	11	Rossi et al. (2006) and Sigsgaard et al. (2001)
*g*_*P*_	Conversion efficiency of predator	indiv/indiv	0.5	Sigsgaard et al. (2001) and Vogel and Moran (2011)
*m*_*P*_	Background mortality of predator	/day	0.01	Thorbek and Topping (2005)
*w*_*N*_	Web site availability per gram native	indiv/g	0.001	Appendix A1
*w*_*I*_	Web site availability per gram garlic mustard	indiv/g	0.1	Appendix A1

## Materials and Methods

### Formulation and assumptions of model

We considered a community as a food web with three trophic levels and modeled dynamics using a system of ordinary differential equations. Our model included four species: an invasive plant (*I*)*,* a native plant (or generalized native vegetation *N*), a shared herbivore (*H*), and a predator (*P*). Model parameters were derived from the literature (Table1).

Both plant species exhibit within-species negative density dependence, compete directly with one another, and are consumed by the shared herbivore (eq. 1 and 2). We assume that the herbivore consumption rate increases linearly with plant density (e.g., a type I or linear functional response). Some herbivores might have upper constraints on their ability to consume plants, due to time or digestive limitations, in which case herbivore feeding rate saturates at high plant density (e.g., a type II functional response). We find, however, that using a type II response does not change model dynamics appreciably compared to a linear functional response (Appendix A3), allowing us to use the simpler linear function to explain species interactions in our model here, without loss of generality. The native and invader plant dynamics are described by:

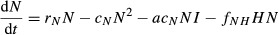
(1)

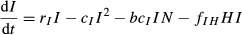
(2)

Within-species negative density dependence is reflected by *c*_*N*_ or *c*_*I*_*,* which is equivalent to the ratio of growth rate to carrying capacity. The strength of interspecific competition is scaled to intraspecific density dependence by *a* or *b,* the impact of the invader on the native and of the native on the invader, respectively. Interspecific competition is reflected by the product of *a* or *b* with *c*_*N*_ or *c*_*I*_ (respectively), where *a* and *b* are the ratio of inter- to intraspecific competition. The herbivore consumes plant biomass at a feeding rate *f*_*NH*_ or *f*_*IH*_.

The herbivore grows by consuming both plant species with a conversion efficiency of plant consumption into herbivore production *g*_*H*_ and experiences losses thro-ugh background mortality, *m*_*H*_, and consumption by a predator (eq. 3). The predator feeding rate is constrained at high herbivore densities, as is typically observed for cobweb spiders (Rossi et al. 2006), so a saturating (type II) functional response is used for the spider. Accordingly, the predator consumes the herbivore with a maximal feeding rate of *f*_*P*_ and a half-saturation constant of *h*_*P*_.

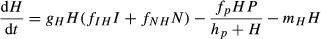
(3)

The predator grows as a function of its herbivore consumption with a conversion efficiency of herbivore mass into predator production *g*_*P*_ and experiences background mortality at a rate *m*_*P*_ (eq. 4). The predator depends upon the two plant species for habitat, so an additional term, (*w*_*N*_*N* + *w*_*I*_*I*), defines the maximum stable population size for the predator as a function of plant densities. This term essentially functions as a carrying capacity for the predator that is directly linked to the plant populations that serve as its habitat.

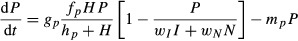
(4)

### Model analysis and parameterization

Small subsets of the model could be solved analytically, and their equilibria are presented in Appendix A2. The full model as well as two subsets could not be solved analytically owing to their complexity. We therefore deployed a numerical analysis complemented by a bifurcation analysis (Appendix A2) to efficiently search parameter space for equilibrium solutions for these three species combinations. All parameters were varied, and continuation software was used to detect bifurcations. Over wide ranges of parameter values shown, the models either converged on a stable equilibrium or exhibited stable limit cycles around the equilibrium (Appendix A2). In order to ensure that the models converged on one equilibrium for the default parameter values regardless of initial species densities, the models were run over a wide range of starting densities for each species under default parameter values (Appendix A2).

We used biologically plausible parameter values (Appendix A1) based on insights from a system that offers the most comprehensive set of parameter values. This system centers around garlic mustard (*Alliaria petiolata*), a ubiquitous invasive herb in eastern deciduous forests that supports elevated densities of cobweb-building spiders in the family Theridiidae (Appendix A1). Garlic mustard may be a strong (*a* > *b*) or weak (*a < b*) interspecific competitor depending on context and the identity of its native competitor (Kalisz et al. 2014; Smith and Reynolds 2014), which is generally true of invasive plant species (Daehler 2003). We set the invader and native to be equal competitors in our model (*a* = *b*) to reflect a middle-ground scenario. Garlic mustard is unpalatable to herbivores (Rodgers et al. 2008), in contrast to many of the native species it competes against, which is reflected in our choice of default feeding coefficients (*f*_*NH*_* *≫ *f*_*IH*_). Theridiidae spiders are known to primarily consume insects, particularly those from the suborder Homoptera (aphids, planthoppers, and leafhoppers) and the order Diptera (flies) (Nyffeler 1999). The herbivorous Homoptera are most likely to directly influence the plant community, so we parameterized our model based on a generalized Homoptera sap-feeding insect.

### Model dynamics

We varied model composition and parameter values in order to evaluate the two mechanisms, as well as to understand how the strength of predator promotion itself (the ability of the invader to support spider webs, *w*_*I*_) influenced model outcomes. We evaluated the potential for predator promotion to initiate a negative feedback that inhibits invasion by comparing results of our full model to six model subsets containing all feasible combinations of the four species (Fig.1). Specifically, we analyzed dynamics for (1) the invasive plant alone; (2) the native plant alone; (3) the invasive plant–native plant couplet; (4) the herbivore-native plant couplet; (5) the herbivore-invasive plant–native plant module; (6) the herbivore–native plant–predator module; and (7) the full predator–herbivore–invasive plant–native plant food web (Fig.1A). Invader–herbivore and invader–herbivore–predator modules were not included because they are not feasible for default parameter values (i.e., the invader cannot support the herbivore alone).

We evaluated the potential for predator promotion to overcome biotic resistance, by varying the relative preference of the herbivore for the native vs. invader by factorially varying *f*_*NH*_ and *f*_*IH*_. This allows us to address the full range of herbivore preference, from preference for the native to preference for the invader to no preference. In order to understand how native community context influenced the importance of predator promotion by invaders, we varied the strength of predator promotion (*w*_*I*_) over gradients of native predator promotion (*w*_*N*_) and herbivore feeding rates (*f*_*NH*_). All model simulations were run in MATLAB using the integrator “ode45.” Simulations were run for 500,000 time steps to eliminate transients, and species densities were averaged over an additional 10,000 time steps to evaluate model results.

### Model stability and robustness

We conducted analytical (subsets 1–4) and numerical (subsets 5–7) analyses that systematically explored a broad range of parameter space for each subset (Appendix A2). Exhaustive analysis of parameter space revealed that for each food web configuration (subsets 1–6 and the full model), densities converge on a single equilibrium for a given set of parameter values regardless of the initial densities of each species (Appendix A2). That is, there is no contingency leading to alternative states within any of the presented subsystems. Model equilibria are stable over a wide range of parameter values (Appendix A2), indicating that the model is robust to realistic variation in parameter values based on different study systems or field measurements.

## Results

### Predator-mediated negative feedback

Predator promotion by an invader could initiate a negative feedback that ultimately inhibits invasion by causing invaders to lose the benefit they gain through enemy escape. This causes the invader to lose its dominance. This result is observed by comparing the equilibria of the subsystems with the full food web. The two plant species coexist stably, with relative densities determined by their relative competitive abilities (Fig.1B, point 3). Consistent with the enemy escape hypothesis, adding herbivores results in a 75% reduction in native plant density and elevates invader density by 37% by releasing the invader from competition with the native (Fig.1B, points 4 and 5). Native density is modestly elevated (a 12% increase) when a predator is introduced into a model without the invasive plant (Fig.1B, point 6). But in the full food web with both plants and the herbivore, adding the predator causes native density to be drastically elevated compared to the case where the predator is absent (270% increase), so that it approaches densities observed in the herbivore-free equilibrium (Fig.1B, point 7). In this case, the invader suffers in the presence of its native competitor and the predator, reducing its equilibrium density dramatically (25%) after the predator is introduced (Fig.1B, points 5 and 7). The predator-promoting invader elevates predator density fourfold (Fig.1C, points 6 and 7) and precipitates a reduction in herbivore density by competing with the native host plant (Fig.1C, points 4 and 5) and by promoting the predator (Fig.1C, points 6 and 7).

### Predator-mediated enemy escape

**Figure 2:**
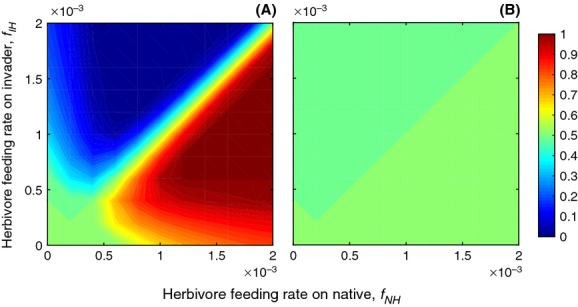
Predator-promoting invaders may overcome biotic resistance to persist even when they are preferred by herbivores. Feeding rates on the native (*f*_*NH*_) and invader (*f*_*IH*_) vary to reflect varying herbivore preferences, where the 1:1 line (not shown) reflects no herbivore preference. Color bar indicates relative density of the invader, where relative density = *I*/(*I*+*N*). (A) When the invader is not a strong predator promoter, the species preferred by the herbivore has a lower relative density. (B) When the invader is a strong predator promoter, top-down control of herbivores makes the role of herbivore preference irrelevant to plant model dynamics.

Predator promotion could release a palatable invader from biotic resistance, but predator-mediated enemy escape is unlikely to drive an invader to high relative abundance. This is because when both invasive and native plants are consumed by herbivores, predators that reduce herbivore density favor both the invader and native simultaneously. Analyzing this mechanism requires manipulating the relative palatability of the invader and native plant species by varying the attack rates (*f*_*NH*_ and *f*_*IH*_, Fig.2). When the invader was not a predator promoter (*w*_*I*_* *= *w*_*N*_* *= 0.0001), the highly palatable plant species preferred by the herbivore had a low relative density, with extinction occurring for the preferred plant species when herbivore attack rates were high (Fig.2A). This includes both the case where the invader is unpalatable (i.e., enemy escape, consistent with our default parameter values) and the opposite case, where the invader is highly palatable (*f*_*NH*_ ≪ *f*_*IH*_; biotic resistance expected). In the latter case, where natives are relatively unpalatable and invaders are palatable, predator promotion can release an invader from biotic resistance brought on by the herbivores. In all cases, when the invader was a predator promoter (*w*_*I*_ = 0.1), both the native and the invader were released from herbivory regardless of which was the preferred forage (Fig.2B). Release from herbivory was dramatic, resulting in nearly equal densities of the native and invader (where the two species are equal competitors) regardless of herbivore preference.

### Context dependence of predator promotion

**Figure 3:**
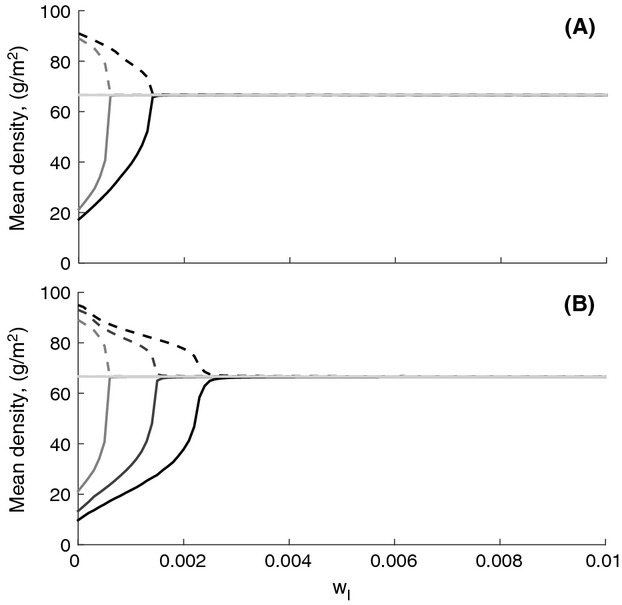
Predator promotion by an invader (reflected as the magnitude of *w*_*I*_) can elevate native plant density (solid lines) and depress invader density (dotted lines) when predator habitat is scarce (low *w*_*N*_) and herbivores feed at native plants high rates (high *f*_*NH*_). (A) Plant densities are shown over a gradient of *w*_*I*_ when natives are weak predator promoters (black lines, low *w*_*N*_ = 0.0001), moderate predator promoters (dark gray lines, *w*_*N*_ = 0.001), or strong predator promoters (light gray lines, high *w*_*N*_ = 0.01). (B) Plant densities are shown over a gradient of *w*_*I*_ when feeding rate of the herbivore on the native is high (black lines, *f*_*NH*_ = 0.004), intermediate (darkest gray lines, *f*_*NH*_ = 0.003), the default value (dark gray lines, *f*_*NH*_ = 0.002), and low (light gray lines, *f*_*NH*_ = 0.001).

The degree to which invaders can alter community dynamics through predator-mediated interactions depends upon several key aspects of the native community context. Predator-promoting structures can only alter predator densities where habitat is limiting, which occurs in cases where habitat quality is low and food availability is high. High values of *w*_*N*_*,* which reflects the native species ability to provide predator habitat, make predator promotion by the invader irrelevant to model dynamics (Fig.3A). Low values of *f*_*NH*_ decrease herbivore growth rate, and therefore predator food availability, making the invader's ability to support predators (*w*_*I*_) less relevant to model dynamics (Fig.3B). Predator promotion by the invader is most likely to boost native plant density when *f*_*NH*_ is high and *w*_*N*_ is low, corresponding to the case where prey are readily available and predator-promoting habitat or substrates are scarce.

## Discussion

Through our theoretical examination of a predator-promoting invader within a food web, we demonstrate that common assumptions about the role of natural enemies determining invasion success or failure may fall apart when invasion is studied in a complete community context. The classic, intuitive expectation is that invaders alter food webs in a way that increases their impact on native communities. However, in the case of the predator-promoting invader, the opposite may be true. Bringing predator promotion into the picture weakens enemy escape as a mechanism promoting invasion and weakens biotic resistance as a mechanism preventing invasion. These results offer one hypothesis as to why these two classic mechanisms – enemy escape and biotic resistance – may receive mixed empirical support (Maron and Vila 2001; Keane and Crawley 2002).

In the case of an unpalatable predator-promoting invader, the advantage gained through enemy escape in the plant–herbivore system is lost when the predator is introduced (Fig.2). Classic theory would suggest that top-down control of herbivores by predators would generally reduce the importance of enemy escape. However, with a predator-promoting invader, the effect of enemy escape is almost completely erased. This is because while the invader directly benefits the predator, it indirectly promotes native plant competitors by reducing their losses to herbivory. This interplay drives a negative feedback loop that promotes the invader's competitor and inhibits its ability to dominate the community.

While many invaders are expected to escape enemies because they are unpalatable, some are palatable forage for herbivores (Maron and Vila 2001; Levine et al. 2004). When invaders are palatable, predator promotion might result in “predator-mediated enemy escape,” where top-down control by predators release invaders from their natural enemies and allows them to dominate a community. But this is not universal. Our model indicates that predator promotion may release an invader from heavy herbivory, but should also release the native species, resulting in little or no relative advantage for the invader. This is reflected in the fact that a system without predator promotion exhibits drastic changes in dynamics as herbivore preference between plants shifts (Fig.2A), while a system with predator promotion is relatively unresponsive to shifting herbivore preference (Fig.2B). Overall, this indicates that while predator promotion could rescue a palatable invader from exclusion through biotic resistance, it would not necessarily drive invader dominance.

Our model is parameterized based on biologically realistic values and predicts an approximately 5× increase predator density with invasion, which falls within the range observed in empirical field studies (Appendix A1). However, the magnitude of the effect of predators on herbivore densities may be attenuated in the field compared to our simplified model. Complexities due to spatial structure, stage structure in herbivore and predator populations, and additional biotic interactions in a larger community (e.g., intraguild predation) may attenuate the magnitude of effects that predators have on herbivores in our full model (subset 7). The model nonetheless advances the hypotheses that predator promotion by invasive can affect invader success. It thereby provides the impetus for new field experiments that test when and how predator and herbivore densities respond to predator-promoting invaders to influence invasion dynamics.

Our model simulates neighbor–neighbor interactions between plants. Thus, the magnitude of predator-prom-oting effects may attenuate with increasing distance from the invader. Spatial heterogeneity cause by invaders aggregating into small patches would likewise be expected to weaken the effect of predator promotion in our model, resulting in less complete top-down control of the herbivore when the predator and invader occur together. This would arise because predator control of the herbivore again drops off with distance from the invader, so predators restricted to large invader monocultures would have reduced impacts. Nevertheless, the nature of the predator promotion effect would be expected to hold: Predator promotion will not benefit an unpalatable invader, but will benefit native plants even if that effect is weakened by distance. In the case of biotic resistance, spatial heterogeneity may have more important qualitative effects, potentially favoring a palatable invader by releasing it from herbivore damage more effectively than the native. This could result in invader dominance through the predicted “predator-mediated enemy escape,” which is not possible when invaders and natives are homogenously distributed.

We note that predator promotion by invaders will have the strongest cascading effects on plant communities when predators are habitat limited. In cases where habitat structure is relatively simple and food availability for predators is high, invaders that promote spiders should shift a system toward top-down control, indirectly increasing native plant density (Fig.3). Such a phenomenon has been observed in agricultural systems, where addition of spider web substrate to a structurally simple environment with high pest densities (a soybean field) reduced crop seedling damage (Halaj et al. 2000a,b). Observations of apparent habitat-limitation in forest contexts, such as the Eastern deciduous forest that garlic mustard invades, are also common (Rypstra 1983; Miyashita and Takada 2007). Such habitat-limitation will be contingent upon the structure of existing native vegetation, making the role of predator promotion in invaders more important in structurally simple habitats.

Identifying the functional traits of invaders that make them predator promoters will aid in predicting which plant species are likely to drive this type of interaction. Invaders in general are likely to increase habitat structure for web spiders simply by forming dense stands of vegetation. However, particularly powerful predator promoters appear to form structures that are unique in the invaded community. These structures may be able to support predators better than co-occurring vegetation, even at equal plant densities, due to vegetation qualities that enhance habitat substrates for predators. For example, invasive knapweed not only dramatically increases web-spider densities, but also doubles per-spider capture rates by allowing them to build larger webs (Pearson 2009). Our model thus underscores the need for a more functional approach to studies of invasion that consider the plant traits that promote habitat for predators (Rypstra 1983; Uetz 1991; Halaj et al. 2000a,b; McNett and Rypstra 2000), and thereby drive community-level predator-mediated feedbacks.

While only a few invaders have been investigated as predator promoters, the fact that elevated spider densities have been observed across widely varying habitat types (forests, grasslands) and plant functional groups (vines, shrubs, herbs) indicates that predator promotion may be quite common among invaders. But, the implications of this predator promotion for communities and for ecosystem functioning remain understudied empirically. Recent observations of predator promotion by invaders, as well as the potential for community-level effects demonstrated by the model presented here, call for further experimental examination of this issue.

## Supporting Information

**Appendix A1.**Explanation of parameter values. **Appendix A2.** Model bifurcation and stability analyses. **Appendix A3.** Analysis of model with saturating functional response in the herbivore.
