# Supplementary material for: Invasive plants may promote predator-mediated feedback that inhibits further invasion
Source: Ecol Evol. 2015 May 25;5(12):2411–9. doi: 10.1002/ece3.1525 (PMC4475373; doi:10.1002/ece3.1525)
Supplement: Supplementary file 1 — Appendix A1.Explanation of parameter values. Appendix A2. Model bifurcation and stability analyses. Appendix A3. Analysis of model with saturating functional response in the herbivore. [file ece30005-2411-sd1.docx]

**Appendix A1**

**Model Parameterization**

We parameterized the model based on invasive garlic mustard (*Alliaria petiolata*) and the cobweb spiders (Theridiidae) it supports. Cobweb spiders consume herbivorous insects, primarily Homoptera (Nyffeler 1999).

The plant growth parameters, *r_N_*, *r_I_*, *c_N_*, and *c_I_*, were determined from a previous experiment in which garlic mustard and commonly co-occuring natives were grown at high densities in community microcosms for a full generation with minimal observed herbivore damage (Smith & Reynolds 2014). Garlic mustard grown alone in pots reached an average biomass of 100 g/m^2^ (dry weight) under typical forest canopy light conditions, based on an average peak biomass per pot of 7 g with a pot surface area of approximately 0.07 m^2^ for a pot with a 15 cm radius. Therefore, we estimate a carrying capacity of 100g/m^2^ when calculating our coefficient of density dependence, *c_I_*, which is equivalent to *r/K* in traditional logistic growth models.

Garlic mustard growth rate, *r_I_*, is estimated based on biomass gains over the course of the above-cited experiment, with consideration for the fact that it would take at least two generations for seed production at a site to be sufficient for garlic mustard to reach its maximum density. From germination of the first generation to maturation of the second, the time to reach carrying capacity would be approximately 1200 days (March year 0 to June year 3), this yields a growth rate ~ *r_I_=*0.1 day^-1^. This is clearly an extreme simplification of the garlic mustard life cycle, which is best modeled using a stage-structured approach. However, our approach is designed to yield reasonable results that can be generalized to other species.

The experiment cited above found native growth rates and maximum densities to be extremely similar to garlic mustard when grown under the same conditions (peak native density was 100 g/m^2^ over the same time period), so carrying capacities and growth rates in the model are the same for the native and the invader.

In our model, interspecific competition was defined by *a* and *b*, which reflect the relative strength of interspecific competition (e.g. effect of I on N) to intraspecific competition (effect of N on N). Therefore, the ‘strength of interspecific competition’ is reflected by the product of *a* x *c_N_* or *b* x *c_I_.* For all values of *a* or *b* less than 1, interspecific competition is weak compared to intraspecific competition, and coexistence between the competitors will occur. Competition coefficients for the two plant species were set to be equal by default (*α*=*β*=0.5). One could argue for setting the native as a stronger competitor (*β* > *α*) or the invader as a stronger competitor (*α*>*β*). Some recent studies that show native species are able to competitively suppress garlic mustard invasion in the absence of other factors (Dornbush & Hahn 2013; Kalisz *et al.* 2014). Other studies have shown strong competitive or allelopathic effects of garlic mustard on some natives (Stinson *et al.* 2007; Smith & Reynolds 2014). The identity of the native species in these studies, as well as other extrinsic factors, likely explain this high level of variation in the literature. To reflect this variation, different competition coefficients could be selected to reflect these dynamics as we gain more information about how different functional groups respond to competition with garlic mustard.

Herbivore feeding and growth parameters were derived from studies in the literature that addressed sap-feeders in the previously recognized Homoptera sub-order. Since aphids are the most studied Homoptera in the literature, many of the studies centered around aphids in forest systems or agricultural systems.

Our herbivore feeding rate parameter, *f_NH_*, was derived from aphids in forest ecosystems, which are known to consume up to 3.4 times their body weight per day in phloem sap (Llewellyn 1970). A reasonable weight estimate for aphids from the agricultural literature is 0.5 mg (Vogel & Moran 2011), so we can estimate phloem consumption of 1.7 g/aphid/day when plants are at carrying capacity. Because this measured is based on fresh weight of sap, we can estimate a dry weight consumption of ~0.17g/aphid/day if sap is 10% sugar by weight, which is a moderate estimate because sap can be highly variable (ranging from <1% to >20% in various agricultural and weedy species, including members of the Brassicaceae (Lohaus *et al.* 1994; Merritt 1996; Caputo & Barneix 1999). We round our parameter estimate to 0.2g/indiv/day as a default feeding rate, which when divided by plant carrying capacity yields *f_NH_*=0.002 indiv^-1^day^-1^. We assume that the herbivore avoids the invasive garlic mustard, which is a very well defended species (Rodgers *et al.* 2008), so we use a feeding rate two orders of magnitude lower of *f_IH_*=0.00002 indiv^-1^day^-1^.

Our estimate of aphid conversion efficiency, *g_H_*, also comes from the tree-dwelling aphid literature. A population-wide estimate of aphid production found that 2444 kcal out of 35,200 kcal consumed go towards aphid production, which yields a mass conversion efficiency of 0.07 (Llewellyn 1972, 1975). Converting mass to aphids based on average weight yields a conversion efficiency of *g_H_*=1.4 indiv/g. Aphid background mortality (*m_H_*) estimates come from the agricultural literature, where young aphids are lost at a rate near 5% in the absence of pesticides (Banks *et al.* 2008).

Our spider parameter estimates come from studies based on spiders in the family Theridiidae or Linyphiidae, two families known to construct webs on garlic mustard that consume similar prey types. An explicit study of spider functional responses found that Theridiidae spiders tend to follow a Type II functional response (Rossi *et al.* 2006). A reasonable maximum feeding rate (*f_P_*) for spiders consuming various Homoptera species (including aphids, leafhoppers, and planthoppers) was found to be 16-23 prey items per day for a Linyphiidae spider(Sigsgaard *et al.* 2001). Half-saturation constants for Theridiidae following at Type II functional response were found to be around 70% of the max feeding rate (Rossi *et al.* 2006). Based on these estimates, we used a default feeding rate of *f_P_*=16 indiv/indiv/day with a half saturation constant (*h_P_*) of 11 indiv. Conversion efficiencies for Linyphiidae spiders consuming Homoptera were found to be 0.5-1 eggs/mg diet (Sigsgaard *et al.* 2001). Since we estimate our Homopteran herbivores to weigh an average of 0.5 mg per individual (Vogel & Moran 2011), this translates to a conversion efficiency of *g_P_*=0.25-0.5 eggs/individual prey item.

Spider background mortality, *m_P_*, is estimated at 0.3% for adult spiders, and 3-4% for eggs and juveniles (Thorbek & Topping 2005). We used a default 1% to reflect a balance between adults and their young without explicitly incorporating stage structure into the model.

The ability of plants to support web spiders, defined by *w_I_* and *w_N_*, was calculated from field surveys (Fig A1). 1m^2^ plots were surveyed across a gradient of garlic mustard invasion at three independent sites. We quantified the number of mature garlic mustard stems as well as the number of active spider webs in each plot. In general, plots where garlic mustard was present at any density supported 5x as many spiders as plots where garlic mustard was absent (5.67 +/- 0.79 spiders/m^2^ and 1.13 +/- 0.55 spiders/m^2^ respectively, calculated from the data shown in Figure A1). At lower invasion densities – where spiders are less likely to be food limited – garlic mustard supports approximately 0.8 spider webs per individual plant (linear regression of data in Figure A1 excluding high garlic mustard densities (>10indiv/m^2^). At an average biomass of 8 g per garlic mustard plant, this equates to 0.8 spiders per 8 g or *w_I_*=0.1. The ability of native vegetation to support spider webs is highly variable based on the species identity. We set *w_N_* to 0.001 as a default value to reflect a species that is a poor substrate for web builders, and explore variation around this value in the manuscript.


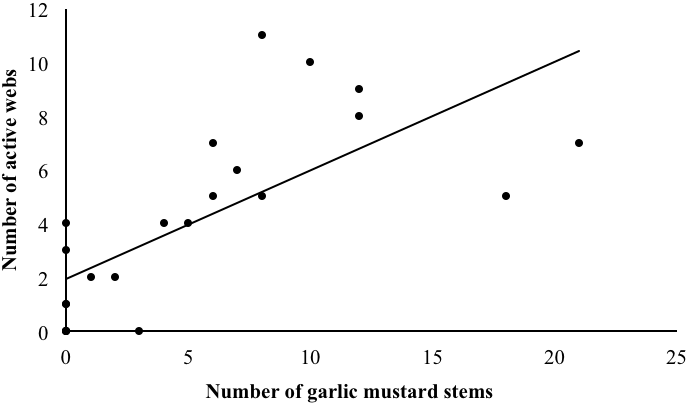


Figure A1. Field surveys showed a positive correlation between the density of garlic mustard and the number of active spider webs across three different sites.

**Appendix A2**

**Model Analysis**

Here we report on the analysis of the equilibria and stability for the full four-species model including two plants, a shared herbivore, and a predator; as well as for selected modular subsets of species (Fig. 1a).

The individual plants (Fig1a, subsets 1 and 2) are stable at their carrying capacities (i.e., I* and N* such that d /dt = 0), where *I*=c_I_*/*r_I_* or *N*=c_N_*/*r_N_*, which is consistent with traditional logistic growth models(Gotelli 2008). The plant-only module (Fig. 1a, subset 3) consists of two plant species with logistic growth and Lotka-Volterra style competition. This classic model has one two-species equilibrium that is stable over all parameter ranges shown in this paper (we keep *α*<1 and *β<*1, to keep interspecific competition weaker than intraspecific competition):

;

This module also has an unstable trivial equilibrium where *I*=*0 and *N*=*0; as well as two unstable one-species equilibria, where one species is extinct and the other species is at its carrying capacity:

; *I*=*0 and ; *N*=*0

These equilibria are equivalent to those from classic Lotka-Volterra competition models(Gotelli 2008).

Stepping up to the three-species module with both plant species and their herbivore (Fig 1a, subset 5), an herbivore with a Type I functional response consumes the two plant species that exhibit logistic growth and compete with each other. This model has one three-species equilibrium that can be solved analytically:

;

;

Populations converge on this equilibrium regardless of starting values (Fig. B1). The behavior of this three-species submodel is examined over a wide range of parameter space through bifurcation analysis (see details under ‘Bifurcation Analysis’ below).

There is an additional two-species equilibrium where the native plant and herbivore co-exist (subset 4):

;

This equilibrium is identical to classical predator-prey models with logistic growth in prey and Type I functional response in the predator, making the equilibrium stable for our parameter values(Gotelli 2008).

An additional species subset is possible where the invader and herbivore co-exist, although this subset is not in Fig. 1a because it is not feasible for our default parameter values, where the invader is unable to support the herbivore:

;

The module consisting of the native, the herbivore, and the spider (Fig 1a, subset 6) has one equilibrium, which must also be solved numerically. Bifurcation analysis was completed for this subset to explore a wide range of parameter space (see below). The model converged on equilibrium densities regardless of starting value (Fig. B2).

The full model (Fig 1a, subset 7) includes four species: the native plant, the invasive plant, the herbivore, and the predator. While equilibria cannot be found analytically for this four-species system, numerical simulations exhibit damped oscillations that converge on one four-species equilibrium for the default parameter values regardless of starting values (Fig B3).

**Bifurcation Analysis**

In order to explore the stability and behavior of the model over a wide range of parameter values, bifurcation diagrams were constructed by varying each parameter while the other parameters were held constant at default values. This analysis was performed for the three-species modules where the invader or spider were absent (Fig. 1a, subsets 5 and 6) as well as for the full four-species model (Fig. 1a, subset 7). The ‘matcont’ numerical continuation software package for MATLAB was used to detect bifurcations(Dhooge *et al.* 2003). For the parameter ranges shown (Table A1), the model reached a stable four-species equilibrium or exhibited stable limit cycles. Analysis was terminated at either end of the parameter range when one species went extinct, at the point that a subcritical Hopf bifurcation occurred and model stability was lost, or when the end of the range of interest for a given parameter was reached. Supercritical Hopf bifurcations occurred above or below the range shown for several parameters, noted in the table. This indicates that beyond these parameter values, the model exhibits stable limit cycles for an extended range of parameter values.

Table B1. Parameter ranges for which model is stable based on bifurcation analysis

| **Parameter** | **Definition** | **Units** | **Default Value** | **Range:**  **Full Model** | **Range: Invader absent** (subset 5) | **Range: Spider absent** (subset 6) |
| --- | --- | --- | --- | --- | --- | --- |
| *r_N_* | Intrinsic growth rate, native plant | day^-1^ | 0.1 | 0.0627-0.198 * | 0.0356-1 | 0.063-1 |
| *c_N_* | Strength of density dependence, native plant | (g/m^2^)^-1^ day^-1^ | 0.001 | **0.0005-0.0015 | 0.00022-0.0032 | 0-0.0016 |
| *f_NH_* | Attack rate of herbivore on native plant | indiv^-1^day^-1^ | 0.002 | 0.0005-0.09 | 0.00073-7.0 | 0.0005-1 |
| *r_I_* | Intrinsic growth rate, garlic mustard | day^-1^ | 0.1 | 0.05-0.176 | n/a | 0.01-0.175 |
| *c_I_* | Strength of density dependence, garlic mustard | (g/m^2^)^-1^ day^-1^ | 0.001 | 0.00057-0.002^+^ | n/a | 0.0006-0.01 |
| *f_IH_* | Attack rate of herbivore on garlic mustard | indiv^-1^day^-1^ | 0.00002 | 0-0.0011 | n/a | 0-0.0004 |
| *a* | Ratio inter- to intra-specific competition (effect of I on N) | -- | 0.5 | 0-0.907 | n/a | 0-0.907 |
| *b* | Ratio inter- to intra-specific competition (effect of N on I) | -- | 0.5 | 0-1 | n/a | 0-1 |
| *g_H_* | Conversion efficiency of herbivore | indiv/g | 1.4 | 0.38-2.93 | 0.67-50 | 0.37-25 |
| *m_H_* | Background mortality of herbivore | day^-1^ | 0.05 | 0-0.188 | 0-0.16 | 0.002-0.18 |
| *f_S_* | Attack rate of spider on herbivore | day^-1^ | 16 | 0.3-75 | 0.26-58.8 | n/a |
| *h_S_* | Half saturation constant for spider | indiv | 11 | 9.6-40 | 0-80 | n/a |
| *g_S_* | Conversion efficiency of spider | indiv/indiv | 0.5 | 0.41-2 | 0.0008-100 | n/a |
| *m_S_* | Background mortality of spider | day^-1^ | 0.01 | 0.000017-0.0357 | 0-1 | n/a |
| *w_N_* | Web site availability per gram native | indiv/gram | 0.001 | 0-0.02 | 0.00004-0.07 | n/a |
| *w_I_* | Web site availability per gram garlic mustard | indiv/gram | 0.1 | 0-0.121  *** | n/a | n/a |

*Supercritical Hopf bifurcation detected at 0.198 ->SLC above this value

**Supercritical Hopf bifurcation detected at 0.0005 ->SLC below this value

+ Supercritical Hopf bifurcation detected at 0.002 -> SLC above this value

***0-0.000365 stable, then supercritical Hopf, SLC 0.000365-0.000512, then stable 0.00513-0.121


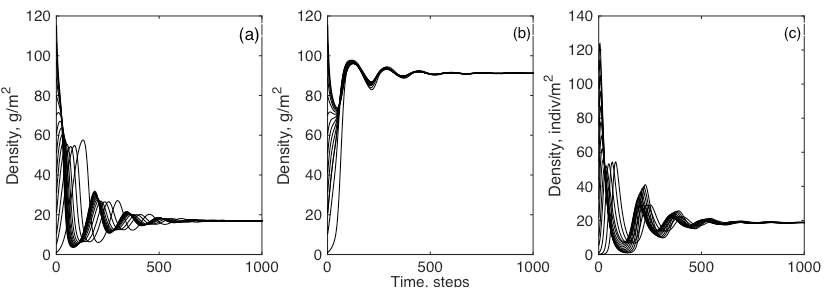


Figure B1. For the three-species module where the spider is absent, densities of the native (a), invader (b), and herbivore (c) converge on one equilibrium regardless of starting values. Simulations were initiated at 14 different starting values for each species, ranging from 1-120 g/m^2^ for plant species and 0.01-30 indiv/m^2^ for the herbivore.


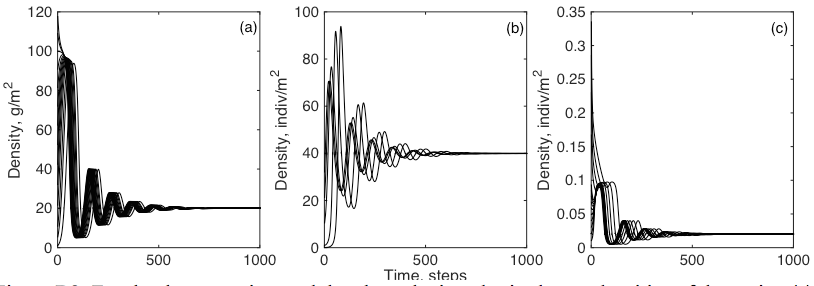


Figure B2. For the three-species module where the invader is absent, densities of the native (a), herbivore (b), and predator (c) converge on one equilibrium regardless of starting values. Simulations were initiated at 14 different starting values for each species, ranging from 1-120 g/m^2^ for the native plant, 0.01-30 indiv/m^2^ for the herbivore, and 0.01-0.5 indiv/m^2^ for the spider.

Figure B3. For the full four-species model, species densities converge on one equilibrium regardless of starting values. Simulations were initiated at 14 different starting values for each species, ranging from 1-120 g/m^2^ for plant species, 0.0001-50 indiv/m^2^ for the herbivore, and 0.0001-50 indiv/m^2^ for the spider. (a) Native and (b) invasive plant densities converge relatively quickly. (c) Herbivore and (d) predator densities appear to converge very quickly in (c) and (d). However, zooming in on the y-axis shows damped oscillations in the herbivore (e) and predator (f) from three starting values for each species (light grey=10, dark gray=0.005, black =0.01) that converge on equilibria over a long time period (600,000 time steps). The solid shapes are actually oscillating densities that appear condensed over the long time scale.

**Appendix A3**

**Considering an herbivore with a Type II functional response**

Here, we consider how adding a saturating (Type II) functional response for the herbivore influences model dynamics.

**Parameterization**

A model with a saturating functional response for the herbivore requires an extra parameter, *h_H_*, the half-saturation constant for the herbivore. We estimate our default *h_H_* to be 60. Feeding rate is parameterized differently for a model with a Type II functional response: units change from indiv^-1^day^-1^ to g/indiv^-1^day^-1^, and our new estimated feeding rate of the herbivore on the native, *f_NH_*, becomes 0.1 g/indiv^-1^day^-1^. To simplify our examination of the role of the Type II functional response, we set up the model so that the herbivore only feeds on the native, consistent with enemy escape, and with our default parameter values for the Type I model where the herbivore attack rate on the invader is minimal (*f_IH_*=0.00002). We note that compared to our standard model, the range of *w_I_* and *w_N_* over which plant densities vary (i.e. the parameter space in which the predator is habitat limited) is constrained, so a reduced default value of *w_N_* is used to illustrate model results.

**Model Analysis**

Here we report on the analysis of the equilibria for the full four-species model including two plants, an herbivore with a saturating functional response, and a predator; as well as for selected modular subsets of species (Fig. 1a).

For the plant species alone and the two-species module consisting of the competing plant species (subsets 1-3, Fig 1a), this version of the model is identical to the primary model with a linear functional response for the herbivore (Appendix A).

We can construct a two-species module of the native plant and its herbivore (subset 4, Fig 1a), which is identical to a Rozensweig-MacArthur predator-prey model(Turchin 2003). In this classical model there is one two-species equilibrium:

$N=\frac{h_{H}m_{H}}{f_{NH}g_{H}-m_{H}}$; $H=\frac{g_{H}h_{H}(f_{NH}g_{H}r_{N}-m_{H}(c_{N}h_{H}+r_{N}))}{(m_{H}-f_{NH}g_{H})^{2}}$

which is stable for the default parameter values in our model, but loses stability when herbivores are relatively efficient (high *g_H_* and/or low *m_H_*) or plant productivity is elevated(Gotelli 2008).

For the three-species module where the spider is absent (subset 5, Fig 1a), an herbivore with a Type II functional response consumes the native plant, and the native and invader compete. This module has one three-species equilibrium:

; ;

This module converges on a stable equilibrium for default parameter values regardless of starting densities (Fig C1).

An additional subset includes the native, its herbivore, and the predator (subset 6, Fig 1a). This subset cannot be solved analytically, but numerical simulations show that densities converge on a stable equilibrium for default parameter values regardless of starting densities (Fig C2).

For the full model including the two plant species, the herbivore, and the predator (subset 7, Fig 1a), bifurcation analysis indicates that for default parameter values, there is one equilibrium that must be solved numerically. Densities converge on this equilibrium regardless of starting values (Fig. C3). We explored variation in parameter values through bifurcation analysis (Table C1).

Mapping the equilibria of the full model and each subset onto plant density axes results in a pattern similar to that presented for the Type I model: the invader loses the advantage granted to it through enemy escape when the spider is present (Fig C4). Bifurcation analysis indicates that this model shows similar behavior to the model with Type I herbivory, although it is constrained to a narrower parameter space (Table C1).

**Bifurcation Analysis**

In order to explore the stability and behavior of the model over a wide range of parameter values, bifurcation diagrams were constructed by varying each parameter while the other parameters were held constant at default values. The ‘matcont’ numerical continuation software package for MATLAB was used to detect bifurcations. For the parameter ranges shown (Table A1), the model reached a stable four-species equilibrium or exhibited stable limit cycles. Analysis was terminated at either end of the parameter range when one species went extinct, at the point that a subcritical Hopf bifurcation occurred and model stability was lost, or when the end of the range of interest for a given parameter was reached. Supercritical Hopf bifurcations occurred above or below the range shown for several parameters, noted in the table. This indicates that beyond these parameter values, the model exhibits stable limit cycles for an extended range of parameter values.

Table C1. Parameter ranges for which model with an herbivore with a saturating functional response is stable based on bifurcation analysis

| **Parameter** | **Definition** | **Units** | **Default Value** | **Range:**  **Full Model** (subset 7) |
| --- | --- | --- | --- | --- |
| *r_N_* | Intrinsic growth rate, native plant | day^-1^ | 0.1 | 0.075-0.199* |
| *c_N_* | Strength of density dependence, native plant | (g/m^2^)^-1^ day^-1^ | 0.001 | 0.0005-0.00133* |
| *f_H_* | Maximum feeding rate of herbivore | g/indiv^-1^day^-1^ | 0.1 | 0.0678-0.127 |
| *h_H_* | Half-saturation constant for herbivore | g/m^2^ | 60 | 48-119 |
| *r_I_* | Intrinsic growth rate, garlic mustard | day^-1^ | 0.1 | 0.05-0.108* |
| *c_I_* | Strength of density dependence, garlic mustard | (g/m^2^)^-1^ day^-1^ | 0.001 | 0.000927-0.108* |
| *a* | Competition coefficient: effect of invader on native | -- | 0.5 | 0-0.8 |
| *b* | Competition coefficient: effect of native on invader | -- | 0.5 | 0.4-1 |
| *g_H_* | Conversion efficiency of herbivore | indiv/g | 1.4 | 0.95-1.55 |
| *m_H_* | Background mortality of herbivore | day^-1^ | 0.05 | 0.044-0.073 |
| *f_P_* | Attack rate of spider on herbivore | indiv^-1^ day^-1^ | 16 | 0.02-400 |
| *h_P_* | Half saturation constant for spider | indiv | 11 | 10.5-70 |
| *g_P_* | Conversion efficiency of spider | indiv/  indiv | 0.5 | 0.47-20 |
| *m_P_* | Background mortality of spider | day^-1^ | 0.01 | 0.000068-0.03312 |
| *w_N_* | Web site availability, native | indiv/  gram | 0.0001 | 0-1** |
| *w_I_* | Web site availability, invader | indiv/  gram | 0.1 | 0-1 |

* Supercritical hopf bifurcation above or below range leads to stable limit cycles

**Region of stable limit cycles within


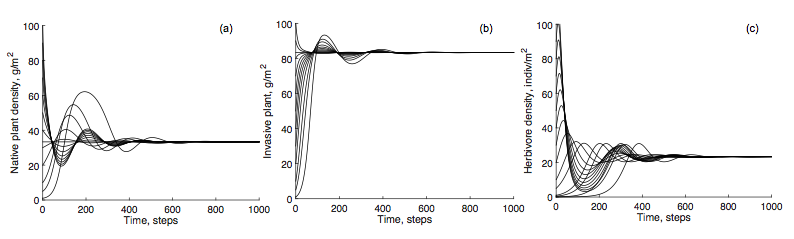


Figure C1. For the three-species module where the spider is absent, densities of the native (a), invader (b), and herbivore (c) converge on one equilibrium regardless of starting values. Simulations were initiated at 12 different starting values for each species, ranging from 1-100 g/m^2^ for plant species and 0.01-100 indiv/m^2^ for the herbivore.


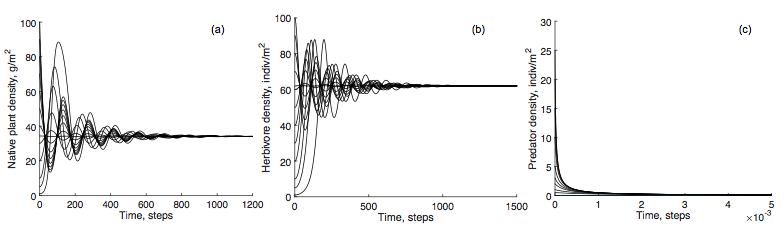


Figure C2. For the three-species module where the invader is absent, densities of the native (a), herbivore (b), and predator (c) converge on one equilibrium regardless of starting values. Simulations were initiated at 12 different starting values for each species, ranging from 1-100 g/m^2^ for the native plant, 0.01-100 indiv/m^2^ for the herbivore, and 0.001-25 indiv/m^2^ for the spider.

Figure C3. For the three-species module where the invader is absent, densities of the native (a), invader (b), herbivore (c), and predator (d) converge on one equilibrium regardless of starting values. Simulations were initiated at 12 different starting values for each species, ranging from 1-100 g/m^2^ for the plant species, 0.01-100 indiv/m^2^ for the herbivore, and 0.001-25 indiv/m^2^ for the spider.

Figure C4. (a) For the case of an herbivore with a saturating (Type II) functional response, feasible subsets (1-6) and the complete food web (7) were analyzed and compared to understand the role of predator-promotion in a system with an invasive plant (I), a native plant (N), and herbivore (H), and a predator (P). Plots show equilibrium densities of (b) the native and invasive plant, and (c) the predator and herbivore for all species subsets. Point 3 (the two plants in competition with one another) and point 7 (the full four-species system with invader, native, herbivore, and predator) overlap significantly in (a), so 7 is indicated by a white diamond. The predator and invader interact to promote elevated density of the native plant (7) compared to subsystems where the invader (4) or predator (6) are absent. The overall pattern is identical to Fig 1., where the herbivore has a linear functional response. The most notable difference is that the density of the predator where present (subsets 6 and 7) is significantly lower when the herbivore has a saturating functional response, although this does not translate to reduced impact on plant and herbivore densities. Note that while the herbivore appears to be extinct in subset 7, it is present at a low density of 0.014 indiv/m^2^.

**Supporting Information References**

Banks, H.T., Banks, J.E., Joyner, S.L. & Stark, J.D. (2008). Dynamic models for insect mortality due to exposure to insecticides. *Mathematical and Computer Modelling*, 48, 316-332.

Caputo, C. & Barneix, A.J. (1999). The relationship between sugar and amino acid export to the phloem in young wheat plants. *Annals of Botany*, 84, 33-38.

Dhooge, A., Govaertz, W. & Kuznetsov, Y.A. (2003). matcont: A matlab package for numerical bifurcation analysis of ODEs. *ACM TOMS*, 29, 141-164.

Dornbush, M.E. & Hahn, P.G. (2013). Consumers and establishment limitations contribute more than competitive interactions in sustaining dominance of the exotic herb garlic mustard in a Wisconsin, USA forest. *Biological Invasions*, 15, 2691-2706.

Gotelli, N.J. (2008). *A Primer of Ecology*. Sinaur Asoociates, Inc., Sunderland, MA 01375-0407 USA.

Kalisz, S., Spigler, R.B. & Horvitz, C.C. (2014). In a long-term experimental demography study, excluding ungulates reversed invader's explosive population growth rate and restored natives. *Proc. Natl. Acad. Sci. U. S. A.*

Llewellyn, M.J. (1970). The ecological energetics of the lime aphid (*Eucallipterus tiliae* L.) and its effect on tree growth. University of Glasgow PhD Thesis.

Llewellyn, M.J. (1972). Effects of lime aphid, *Euacallipterus-tiliae* L (Aphididae) on growth of lime *Tilia* X *vulgaris* Hayne. I. Energy requirements of the aphid population. *Journal of Applied Ecology*, 9, 261-&.

Llewellyn, M.J. (1975). Effects of lime aphid, *Euacallipterus-tiliae* L (Aphididae) on growth of lime *Tilia X vulgaris* Hayne. 2. The primary production of saplings and mature trees, energy drain imposed by aphid populations, and revised standard deviations of aphid population energy budgets *Journal of Applied Ecology*, 12, 15-23.

Lohaus, G., Burba, M. & Heldt, H.W. (1994). Comparison of the contents of sucrose and amino-acids in the leaves, phloem sap and taproots of high and low sugar-producing hybrids of sugar-beet (*Beta-vulgaris L*). *Journal of Experimental Botany*, 45, 1097-1101.

Merritt, S.Z. (1996). Within-plant variation in concentrations of amino acids, sugar, and sinigrin in phloem sap of black mustard, *Brassica nigra* (L) Koch (Cruciferae). *J. Chem. Ecol.*, 22, 1133-1145.

Nyffeler, M. (1999). Prey selection of spiders in the field. *Journal of Arachnology*, 27, 317-324.

Rodgers, V.L., Stinson, K.A. & Finzi, A.C. (2008). Ready or not, garlic mustard is moving in: *Alliaria petiolata* as a member of eastern North American forests. *Bioscience*, 58, 426-436.

Rossi, M.N., Reigada, C. & Godoy, W.A.C. (2006). The effect of hunger level on predation dynamics in the spider *Nesticodes rufipes*: a functional response study. *Ecological Research*, 21, 617-623.

Sigsgaard, L., Toft, R. & Villareal, S. (2001). Diet-dependent fecundity of the spiders *Atypena formosana* and *Pardosa pseudoannulata*, predators in irrigated rice. *Agric. For. Entomol.*, 3, 285-295.

Smith, L.M. & Reynolds, H.L. (2014). Light, allelopathy, and post-mortem invasive impact of garlic mustard on native forest understory species. *Biological Invasions*, 16, 1131-1144.

Stinson, K., Kaufman, S., Durbin, L. & Lowenstein, F. (2007). Impacts of garlic mustard invasion on a forest understory community. *Northeastern Naturalist*, 14, 73-88.

Thorbek, P. & Topping, C.J. (2005). The influence of landscape diversity and heterogeneity on spatial dynamics of agrobiont linyphiid spiders: An individual-based model. *Biocontrol*, 50, 1-33.

Turchin, P. (2003). *Complex Population Dynamics: A Theoretical/Empirical Synthesis*. Princeton University Press, Princeton, NJ.

Vogel, K.J. & Moran, N.A. (2011). Sources of variation in dietary requirements in an obligate nutritional symbiosis. *Proceedings of the Royal Society B-Biological Sciences*, 278, 115-121.
